# CIGB-300 Peptide Targets the CK2 Phospho-Acceptor Domain on Human Papillomavirus E7 and Disrupts the Retinoblastoma (RB) Complex in Cervical Cancer Cells

**DOI:** 10.3390/v14081681

**Published:** 2022-07-30

**Authors:** Ailyn C. Ramón, Om Basukala, Paola Massimi, Miranda Thomas, Yasser Perera, Lawrence. Banks, Silvio E. Perea

**Affiliations:** 1 Molecular Oncology Group, Department of Pharmaceuticals, Biomedical Research Division, Center for Genetic Engineering and Biotechnology (CIGB), Havana 10600, Cuba; ailyn.ramon@cigb.edu.cu (A.C.R.); ypereranegrin@ccbjic.com (Y.P.); 2 Tumor Virology Group, International Centre for Genetic Engineering and Biotechnology (ICGEB), AREA Science Park, 34149 Trieste, Italy; basukala@icgeb.edu.cu (O.B.); paola.massimi@icgeb.org (P.M.); miranda.thomas@icgeb.org (M.T.); 3 China-Cuba Biotechnology Joint Innovation Center (CCBJIC), Yongzhou Zhong Gu Biotechnology Co., Ltd., Lengshuitan District, Yongzhou 425000, China

**Keywords:** HPV E7, protein kinase CK2 inhibitor, CIGB-300, pRB

## Abstract

CIGB-300 is a clinical-grade anti-Protein Kinase CK2 peptide, binding both its substrate’s phospho-acceptor site and the CK2α catalytic subunit. The cyclic p15 inhibitory domain of CIGB-300 was initially selected in a phage display library screen for its ability to bind the CK2 phospho-acceptor domain ofHPV-16 E7. However, the actual role of this targeting in CIGB-300 antitumoral mechanism remains unexplored. Here, we investigated the physical interaction of CIGB-300 with HPV-E7 and its impact on CK2-mediated phosphorylation. Hence, we studied the relevance of targeting E7 phosphorylation for the cytotoxic effect induced by CIGB-300. Finally, co-immunoprecipitation experiments followed by western blotting were performed to study the impact of the peptide on the E7–pRB interaction. Interestingly, we found a clear binding of CIGB-300 to the N terminal region of E7 proteins of the HPV-16 type. Accordingly, the in vivo physical interaction of the peptide with HPV-16 E7 reduced CK2-mediated phosphorylation of E7, as well as its binding to the tumor suppressor pRB. However, the targeting of E7 phosphorylation by CIGB-300 seemed to be dispensable for the induction of cell death in HPV-18 cervical cancer-derived C4-1 cells. These findings unveil novel molecular clues to the means by which CIGB-300 triggers cell death in cervical cancer cells.

## 1. Introduction

Cervical cancer is one of the most common causes of cancer-related death for women across the world. Human papillomavirus (HPV) is the causative agent of cervical cancer and a large number of other human malignancies [1,2]. Although the prevalence and mortality of cervical cancer have been reduced thanks to prophylactic vaccines and earlier diagnosis, cervical cancer is still a global concern [3]. Prophylactic vaccines prevent HPV infection and consequently prevent HPV-associated cancers; however, they have no effect on pre-existing HPV infections and HPV-associated lesions [4]. Current therapeutic strategies include surgical removal of the lesion and radiotherapy plus cisplatin-based chemotherapy; however, these do not specifically target the oncogenic properties of HPV, and therefore lesion recurrence can occur [5]. Thus, the scientific community is focused on improving the current therapeutic approaches by combining strategies or by searching for novel agents effective at treating HPV-associated cancer.

HPV is a small non-enveloped DNA virus that infects keratinocytes in the differentiating epithelium of the skin and mucosae. The high-risk HPV-16 and HPV-18 subtypes are responsible for around 70% of all cervical cancer cases, whereas the low-risk types, including HPV-6 and HPV-11, cause benign genital warts (condylomas) [6,7]. The viral proteins E6 and E7 are well-known HPV oncogenes that play a critical role in cellular growth control pathways, which can also cause cells to undergo transformation [6]. Both viral proteins enhance tumorigenesis and thus constitute relevant targets for therapeutic intervention in HPV-induced malignancy. E6 triggers part of its oncogenic activity by inducing the degradation of the p53 tumor suppressor, as well of a number of PDZ domain-containing proteins. E7 is also a relevant target for HPV-positive cervical cancer therapy [8,9]. E7 targets the pRB family of tumor suppressors for proteasome-mediated degradation, facilitating the expression of the DNA synthesis machinery in differentiated keratinocytes [10]. Other E7-interacting partners with roles in carcinogenesis include transcriptional regulators, such as the TATA box-binding protein (TBP), p300/CBP and E2F [11].

Phosphorylation is the major post-translational modification of E7 that affects some of these interactions. In particular, the presence of a CK2 phospho-acceptor site within the CR2 domain of E7 seems to enhance E7 interaction with different cellular target proteins, thereby increasing the ability of E7 to enhance cell proliferation and, potentially, malignant transformation [10,12,13]. Recently, substitution of CK2 phospho-acceptor sites on the E7 protein by non-phosphorylable residues (i.e., S32A and S34A) was shown to produce slow-growing cells with reduced invasion capacity in Matrigel-based assays, thus confirming the important role of CK2-mediated phosphorylation of E7 for the maintenance of the cancer phenotype once the tumor is established [14].

The peptide CIGB-300 is a CK2 inhibitor with a dual mechanism; it binds to conserved phospho-acceptor sites on substrates, as well as directly targets the enzyme [15,16,17]. Initially, the peptide was selected from a phage display peptide library by its ability to bind the CK2 phospho-acceptor domain of HPV-16 E7 and block phosphorylation [15]. CIGB-300 inhibits cell proliferation and induces apoptosis in cervical cancer cell lines and halts tumor growth in an HPV-16 syngeneic murine tumor animal model [15,18,19]. In the clinical setting, it has been shown that CIGB-300 is safe and well tolerated in cancer patients and healthy subjects [20,21,22]. Specifically, phase I/II studies in locally advanced cervical cancer patients demonstrated clinical effects of intratumoral injections of CIGB-300 [23,24,25,26]. Despite the wide range of preclinical and clinical evidence of CIGB-300 antitumoral activity in this therapeutic niche, the mechanism through which the peptide affects cervical cancer cells is not fully elucidated. Previously, investigation of the molecular and cellular events leading to apoptosis in CIGB-300-treated cancer cell lines suggested B23/Nucleophosmin as a major target [18]. However, downregulation of B23/Nucleophosmin in Acute Myeloid Leukemia cells only partially recapitulated the cytotoxic effect of the peptide, suggesting the existence of other molecular targets [17,27].

For the first time, we explored here the putative interaction of CIGB-300 with E7 oncoprotein in the cellular context, the relevance of the targeting, and its contribution to CIGB-300 cytotoxic effect. Our results demonstrate that the effect of CIGB-300 on CK2-mediated phosphorylation of E7 does not fully support its cytotoxic effect on cervical cancer. However, the interaction of the peptide with E7 impairs E7 ability to bind the pRB tumor suppressor. Altogether, the data presented here provide further molecular evidence as to the means by which CIGB-300 induces cell death in cervical cancer.

## 2. Materials and Methods

### 2.1. Cell Culture

The CaSki (CRL-1550^TM^), HeLa (CCL-2^TM^), SiHa (HTB-35^TM^), C4-1(CRL-1594^TM^) and HEK293 (CRL-1573^TM^) cell lines were obtained from the American Type Culture Collection (ATCC) and maintained in Dulbecco’s modified Eagle’s medium (DMEM) (Gibco, Carlsbad, CA, USA), supplemented with 10% fetal bovine serum (Gibco, Carlsbad, CA, USA), penicillin–streptomycin (100 U/mL) and glutamine (300 μg/mL) (Gibco, Carlsbad, CA, USA).

### 2.2. Compounds

CIGB-300 was dissolved in PBS at room temperature for 5 min to obtain a 10 mM stock. For each experiment, a freshly made stock was used. CX-4945 was obtained from SelleckChem (Munich, Germany) and was resuspended in dimethyl sulfoxide (DMSO) to obtain a 10 mM stock solution. The drugs were diluted directly into the growth media just prior to use.

### 2.3. Plasmid Constructs

The plasmids expressing glutathione-S-transferasa (GST)-E7 from HPV subtypes 11, 16, and 18, GST alone, and the GST-fused N-terminal and C-terminal halves of HPV-16 E7 were described previously [28,29].

The plasmids pCMV:HPV-16 E7-FLAG-HA and pGEX:HPV-18E7 were a kind gifts from Karl Münger [30].

### 2.4. Cell Transient Transfection

HEK293 cells were seeded in appropriate dishes and incubated for ~24 h until reaching a confluency of 60–70%. The medium was then changed, and a transfection solution containing the respective DNAs (empty pCMV vector and pCMV FLAG-HA-tagged HPV-16 E7) in Tris-EDTA (TE) buffer and CaCl_2_ (Solution A) was prepared and added dropwise to Solution B (2 × HBS), followed by 30 min of incubation at room temperature. The transfection mix was then added to the appropriate dish. Transfected cells were incubated at 37 °C for 48 h in a humidified CO_2_ incubator and then harvested for further analysis. Solutions A and B were prepared as described [31].

### 2.5. Cell Viability Assay and Drug Treatments

Cell viability was determined by the XTT assay. Briefly, 20,000 C4-1 wildtype and mutant cells per well were seeded in flat-bottomed 96-well plates in DMEM medium with 10% fetal bovine serum (FBS) and incubated overnight at 37 °C, 5% CO_2_. Then, a series of serial dilutions (1:2) of CIGB-300 (31.25–500 μM) and CX-4945 (3.125–50 µM) were added in triplicate. After 48 h, 50 µL of XTT labeling mixture (5 mL XTT labelling reagent + 0.1 mL electron coupling reagent) (Roche, Basel, Switzerland) was added to each well, and the cells were further incubated for 4 h at 37 °C. Following the incubation period, absorbance at 490 nm was read using an ELISA plate reader (BioRad, Watford, UK). The percentage of cell viability was calculated using the following formula: Cell viability (%) = ((OD of treated cells)/(OD of cell control)) × 100. The half-cytotoxic concentration (CC_50_) was estimated from the fitted dose–response curves using the CalcuSyn software (Biosoft, Cambridge, UK).

### 2.6. Production and Purification of GST-Fusion Proteins

The expression plasmids were transformed into *E. coli* strain BL21. Bacteria containing the GST constructs were grown in Luria Broth (LB) culture media with 75 µg/mL of Ampicillin (Sigma, St. Louis, MO, USA) overnight at 37 °C and then 1 h at 37 °C. After incubation, Isopropyl-β-d-thiogalactopyranoside (IPTG) was added to 1 mM, and the bacteria were further incubated for 3 h at 37 °C in a shaker. Following the IPTG treatment, the bacteria were centrifuged at 5000 rpm for 5 min, and the pellets were lysed in 5–10 mL of 1X PBS containing 1% Triton X-100, then sonicated on ice for 30 s at 80% amplitude. The lysates were clarified by centrifugation, and the supernatants were collected and incubated with glutathione-conjugated agarose beads on a rotating wheel overnight at 4 °C. The GST-fusion protein-conjugated beads were centrifuged at 2000 rpm for 1 min, then the supernatant was removed, and the beads were washed three times with 1X PBS containing 1% Triton X-100. The GST-fusion protein-containing beads were stored with 20% glycerol at −20 °C, and protein purity was monitored by SDS-PAGE and Coomassie blue staining.

### 2.7. In Vitro Binding Assay Using GST-Fusion Proteins

Direct binding assays were performed by incubating biotin-tagged CIGB-300 (100 μM) with GST-fusion proteins bound to glutathione-agarose for 1 h at 4 °C. After extensive washing with PBS containing 1% NP-40, the bound peptide was analyzed by SDS-PAGE with the appropriate antibody and autoradiography.

### 2.8. In Vitro/In Vivo Pull-Down Assay

The E7-CIGB-300 interaction was evaluated by in vitro/in vivo pull-down followed by western blot experiments. For the in vitro pull-down, the cells were seeded in 175 cm dishes and incubated to a confluency of 60–70%. Afterward, the cells were washed, collected, and lysed in lysis buffer RGMT (50 mM HEPES pH 7.4, 150 mM NaCl, 1 mM MgCl_2_, 1 mM NaF, 1% Triton-x-100, plus a protease inhibitor cocktail I [Calbiochem, San Diego, CA, USA]). The cellular lysates were cleared by centrifugation, and 225 µL of total protein extract was incubated with biotin-tagged CIGB-300 (100 μM) or biotin-tagged scrambled peptide (10 mg/mL) for 2 h at 4 °C, then added to 20 µL of pre-equilibrated streptavidin-coated magnetic Sepharose beads (Cytiva, Marlborough, MA, USA) and incubated 1 h at 4 °C. The beads were then collected using a magnetic rack and extensively washed with cold RGMT. The streptavidin beads bound to CIGB-300-interacting proteins were resuspended directly in 2X SDS-PAGE sample buffer, resolved on a 15% SDS-PAGE gel and analyzed by western blot.

For in vivo pull-down assays, the cells were treated with biotin-tagged CIGB-300 (200 μM) or PBS for 30 min at 37 °C in 5% CO_2_. Subsequently, the cells were collected, and a pull-down assay was conducted, as above. Proteins bound to streptavidin magnetic beads were eluted, resolved on a 15% SDS-PAGE gel and analyzed by western blot, as described below.

### 2.9. In Vitro/In Vivo Phosphorylation Assay

For in vitro phosphorylation, purified GST-fusion proteins were incubated with CK2 enzyme (New England Biolabs NEB, Ipswich, MA, USA) in 20 μL kinase buffer (20 mM Tris-HCI [pH 7.5], 5 mM MnCl_2_) in the presence of 10 nM ATP for 15 min at 30 °C. CIGB-300 was incubated with the GST-fusion protein on a rotating wheel for 1 h before the phosphorylation reaction, while CX-4945 was added before the enzyme. After extensive washing with a kinase wash buffer (20 mM Tris-HCI [pH 7.5], 5 mM MnCl_2_, 0.1% NP-40), the GST-fusion proteins were subjected to SDS-PAGE and western blot analysis using an anti-phospho-16-E7 antibody.

HEK293 cells were seeded onto 10 cm dishes and co-transfected with 3 μg of FLAG-HA-tagged HPV-16 E7 or empty vector. After 24 h, the cells were treated with 25 µM of CX-4945 for 2 h and 200 µM of CIGB-300 for 30 min, 2 h and 6 h at 37 °C. The cells were harvested and analyzed by western blot.

### 2.10. Immunoprecipitation Assay

For immunoprecipitation, HEK293 cells were transfected with FLAG-HA-tagged pCMV HPV-16 E7 plasmid and empty vector. After 48 h, the cells were treated with 25 µM CX-4945 for 2 h and 200 µM CIGB-300 for 30 min at 37 °C. The cells were then harvested using a lysis buffer (50 mM HEPES pH7.4, 150 mM NaCl, 1 mM MgCl_2_, 1 mM NaF, 1% Triton-x-100, protease inhibitor cocktail I [Calbiochem, San Diego, CA, USA) and centrifuged at 14,000 rpm for 10 min. The supernatant was incubated with 30 μL of monoclonal anti-HA agarose beads (Sigma, St. Louis, MO, USA) on a rotating wheel at 4 °C for 2 h. After incubation, the samples were washed with the lysis buffer. The immunoprecipitates were then run on SDS PAGE gels and analyzed by western blot.

### 2.11. Proteins Detection by Western Blot and Antibodies

For western blot of whole cell extracts, the cells were harvested and lysed directly in 2X SDS-PAGE sample buffer. Whole cell extracts or proteins extracts from the pull-down and immunoprecipitation assays were then electrophoresed on SDS-polyacrylamide gels and transferred to 0.22 μm nitrocellulose membranes (Amersham, UK). The membranes were blocked in 5% non-fat milk powder dissolved in TBST (20 mM Tris-HCl pH 7.5, 150 mM NaCl, 0.1% Tween-20). The membranes were then probed for different proteins using the appropriate primary antibodies, i.e., mouse monoclonal anti-HA (1:500; Roche, Basel, Switzerland), mouse monoclonal anti-HPV-16 E7 (1:200), mouse monoclonal anti-HPV-18 E7 (1:200) from Santa Cruz Biotechnology (Dallas, TX, USA), mouse monoclonal anti-Rb (1:1000) (G3-245; BD Pharminge, San Diego, CA, USA), mouse monoclonal anti-α-tubulin, mouse monoclonal anti-HA-peroxidase (clone HA-7), and streptavidin–HRP (1:3000) (Dako-Cytomation, Glostrup, Denmark). The HPV-16 E7 pS31/S32 peptide antibody generated by Eurogentec was described previously [14]. Incubation with the primary antibodies was followed by incubation with the respective HRP-conjugated anti-mouse or anti-rabbit secondary antibodies (1:2000; Dako-Cytomation, Glostrup, Denmark). Detection of peroxidase activity was performed by using the Amersham ECL western blot detection kit (GE Healthcare, Chicago, IL, USA).

### 2.12. Statistical Analysis

For the quantification of protein levels from the western blots, the band intensities were measured using Image J software. Differences between groups were determined using one-way ANOVA, followed by Dunnett’s multiple comparison test. The analyses were condicted using GraphPad Prism (v6.01) software (GraphPad Software, Inc., San Diego, CA, USA). A *p* value below 0.05 was considered statistically significant.

## 3. Results

### 3.1. CIGB-300 Interacts with E7 Protein In Vitro

**Figure 1:**
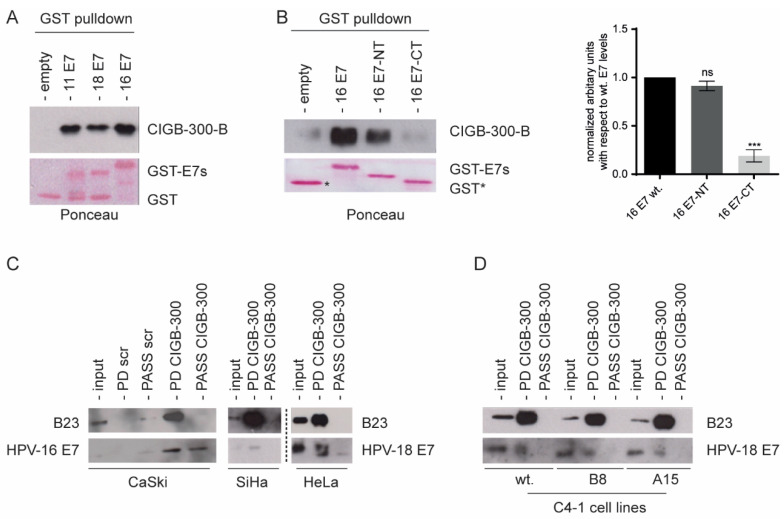
In vitro physical interaction of CIGB-300 with E7 protein. Western blot analysis of in vitro pull-down fractions using CIGB-300 and scrambled peptide, both conjugated to biotin as a bait to capture interacting proteins. GST pull-down was carried out using the purified GST-tagged E7 from HPV-11, HPV-16, HPV-18 (**A**) and HPV-16 E7 N-terminus and HPV-16 E7 C-terminus (**B**). The GST fusion proteins were incubated 1 h with CIGB-300, then the CIGB-300-E7 interaction was resolved by 20%-SDS-PAGE and analyzed by western blot. The top panels show the immunoblot analysis for CIGB-300 using an anti-streptavidin antibody, and the lower panels show the Ponceau staining for different GST-fusion proteins. The position of empty GST (*) is shown. Relative densitometry analysis values of bands normalized to HPV-16 E7 are indicated. In vitro pull-down was performed with cellular lysates from CaSki, SiHa, HeLa (**C**), as well as C4-1 wildtype and mutant cells (**D**) incubated 1 h with CIGB-300 (100 μM). Subsequently, 20 µL of streptavidin magnetic beads was added to each reaction, and CIGB-300-interacting proteins were eluted, resolved on 15%-SDS-PAGE and subjected to western blot. The scrambled control peptide sequence is a stretch of 10 random amino acids. Input: cellular extract. PD: pull-down fractions. PASS: flow-through fraction. Results from (**B**) represent means from three independent experiments, while those from (**A**,**C**,**D**) are representative of two independent experiments. Statistically significant differences are represented as *** *p* < 0.001 determined using one-way ANOVA followed by Dunnett’s post-test; ns indicates non-significant.

We first investigated the putative physical interaction between the CIGB-300 peptide and the E7 viral protein from both high-risk HPV-16/-18 and low-risk HPV-11. We conducted in vitro pull-down experiments using biotinylated CIGB-300 and GST-fusion proteins. The peptide was incubated with GST-tagged HPV-11, -18, or -16 E7, or empty GST as negative control, followed by immunoblot analysis. The in vitro interaction was detected using an anti-streptavidin antibody recognizing the biotinylated peptide. The data in Figure 1A show that CIGB-300 interacts with both HPV-16 and HPV-18 E7 oncoproteins, as well as with HPV-11 E7. To look for the HPV-16 E7 region targeted by CIGB-300, we repeated the peptide interaction assay using the GST-tagged HPV-16 E7 N-terminus and the GST-tagged HPV-16 E7 C-terminus. Consistent with the location of the CK2 phospho-acceptor domain, CIGB-300 preferentially bound to the conserved N terminal part of the E7 protein (Figure 1B). Similarly, binding of the peptide to the HPV-16 and -18 E7 proteins was detected in lysates derived from CaSki, SiHa and HeLa cells, while no binding was detected with the scrambled peptide, further confirming the specific interaction of the peptide with E7 (Figure 1C). Such binding occurred independent of the phosphorylation status of E7, since the peptide clearly interacted with E7 from C4-1 cells containing a mutated CK2 phospho-acceptor site (Figure 1D).

### 3.2. CIGB-300 Interacts with E7 Protein In Vivo

**Figure 2:**
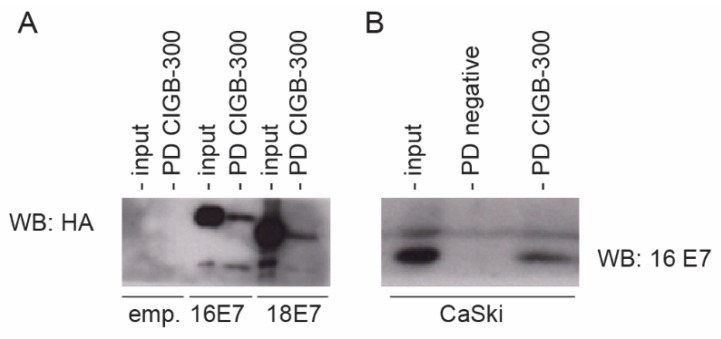
In vivo interaction of CIGB-300 with E7 protein. FLAG-HA-tagged HPV-16 E7 was overexpressed in HEK293 (**A**) and CaSki (**B**) cells. The cells were treated with biotin-tagged CIGB-300 (200 μM) for 30 min and then processed as described in “Materials and Methods”. CIGB-300-interacting proteins were separated by SDS-PAGE and immunoblotted using anti-HA-tag and anti-HPV-16 E7 antibodies for HEK293 and CaSki, respectively. PD: pull-down fractions; NC: negative control (cells incubated with the empty vector). Results are representative of three independent experiments.

Having shown that CIGB-300 interacts with E7 in vitro, we wanted to assess the interaction in a relevant cellular context. Accordingly, we conducted in vivo pull-down assays using HEK293 cells transfected with constructs expressing FLAG-HA-tagged HPV-16 E7 or HPV-18 E7. The data shown in Figure 2A clearly indicate that the CIGB-300 peptide binds to both HPV-16 and HPV-18 E7, confirming the results obtained in vitro. Additionally, we explored the 16 E7–CIGB-300 interaction in the cervical cancer-derived cell line CaSki and obtained similar results (Figure 2B).

### 3.3. Inhibition of E7 Phosphorylation Is Not Essential for CIGB-300 Cytotoxicity in Cervical Cancer Cells

**Figure 3:**
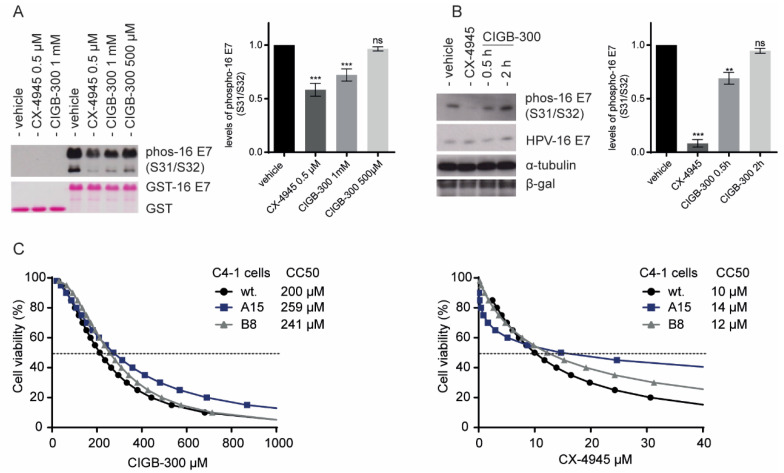
Impact of targeting HPV-16 E7 CK2-mediated phosphorylation on the cytotoxic effect of CIGB-300. (**A**). In vitro phosphorylation assay, using purified GST-HPV-16 E7 fusion proteins incubated with the purified CK2 enzyme, in the presence of ATP and CK2 inhibitors. The samples were analyzed by western blot using an antibody specific for phosphorylated HPV-16 E7 (S31/S32) (top panel). The bottom panel shows the Ponceau-stained membrane, indicating the total levels of GST-fusion E7 protein and GST control. (**B**). In vivo phosphorylation assay using E7-overexpressing HEK293 cells. The cells were treated with the CK2 inhibitor CIGB-300 (200 µM) and CX-4945 (25 µM) for 30 min and 2 h, respectively. The cells were then harvested directly in 2X sample buffer, resolved on 15%-SDS-PAGE and subjected to western blot analysis to identify phosphorylated E7 and total protein levels with the anti-HA antibody. β-gal was employed as a loading control. Relative densitometry analysis of p S31/32 HPV-16 E7 bands normalized to vehicle are indicated (**C**). Effect of CIGB-300 on the viability of wildtype and mutant C4-1 cells, using an XTT assay. The indicated cervical cancer cell lines were cultured for 48 h with increasing concentrations of CIGB-300 and CX-4945. CC_50_ was estimated from the fitted dose–response curves based on treatment with five CK2 inhibitor concentrations, as determined by the cell viability assay. Results in (**A**,**B**) are shown as means ± SD from three independent experiments, while those in **C** are representative of three independent experiments (three replicates each). Statistically significant differences are represented as ** *p* < 0.01 and *** *p* < 0.001, determined using one-way ANOVA followed by Dunnett’s post-test; ns indicates non-significant.

To investigate the effect of CIGB-300 on the CK2-mediated phosphorylation of E7, we conducted western blot analysis using GST-fusion proteins and total cell extracts derived from HEK293 cells overexpressing FLAG-HA-tagged HPV-16 E7. Using a specific anti-HPV-16 E7(S31/S32) antibody, we confirmed the in vitro inhibitory effect of CIGB-300 on E7 phosphorylation (Figure 3A). Accordingly, CIGB-300 inhibited nearly 40% of in vivo E7 phosphorylation after 30 minutes of treatment (Figure 3B). The CX-4945 compound was included in this assay as a reference for the global inhibition of CK2-mediated mediated phosphorylation in the cells (Figure 3B).

Having demonstrated that CIGB-300 can inhibit CK2-mediated phosphorylation of the S31/S32 residues of E7, we explored the relevance of such inhibition for the cytotoxic effect of CIGB-300, using C4-1 cells expressing E7 mutated at the CK2 phospho-acceptor site. These cells were generated by a genome-editing approach in which the S32/S34 amino acid residues in E7 were changed to A32/A34, thereby impairing E7 susceptibility to phosphorylation [14]. The impact of CIGB-300 on the viability of wildtype C4-1 cells and the mutant clones A15 and B8 was assessed by the XTT assay. Figure 3C shows the corresponding dose–response curves in the presence of CIGB-300 and CX-4945. C4-1 cells, including wildtype and both mutant clones A15 and B8, exhibited a similar response to the treatment with the CK2 inhibitors and consistently presented no differences in viability at clinically relevant doses of the inhibitors. Wildtype C4-1 cells showed a half-cytotoxic concentration, CC_50_, of 200 µM, whereas the mutant clones showed a mean value of 259 µM; however, no significant sensitivity toward CIGB-300 cytotoxic effect was displayed. Our results indicate that the ability to target E7 phosphorylation is not an essential molecular event in the cell death induced by CIGB-300 in cervical cancer cells.

### 3.4. CIGB-300 Affects the HPV-16 E7–pRB Complex Formation

**Figure 4:**
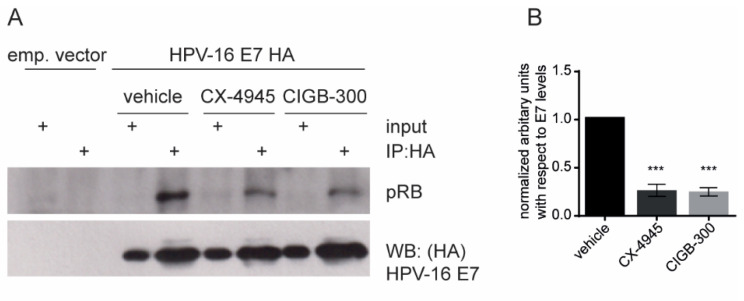
Effect of inhibiting CK2 activity on the E7–pRB interaction. HEK293 cells were transfected with the empty pCMV vector or with pCMV:FLAG-HA-HPV-16 E7. The cell lysates were immunoprecipitated using an anti-HA antibody immobilized on agarose beads. (**A**). The immunoprecipitated complexes were then washed with lysis buffer and analyzed by western blot for pRB and total E7. The panel shows the protein inputs and the results of the immunoprecipitation. (**B**). Quantification of the levels of immunoprecipitated pRB with respect to the levels of E7 in the presence of CK2 inhibitors. Data are shown as means ± SD, *n* = 3. Statistically significant differences between vehicle and drug treatment are indicated with *** *p* < 0.001, determined using one-way ANOVA followed by Dunnett’s post-test.

CIGB-300 was initially designed to bind the CK2 phospho-acceptor domain of HPV-16 E7 and, as we confirmed here, it was shown to bind preferentially to the N-terminal region of E7, near the pRB binding domain (Cys24). To determine whether the peptide disrupts the binding of HPV-16 E7 to pRB, we performed immunoprecipitation analysis of HEK293 cells transfected with constructs expressing FLAG-HA-tagged HPV-16 E7 or the empty vector as a negative control. We observed a clear decrease of the pRB signal in the HPV-16 E7 immunoprecipitated fraction after treatment with either the known CK2 inhibitor CX-4945 or CIGB-300 (Figure 4). This result indicates that CIGB-300 can disrupt the binding between the HPV-16 E7 and pRB protein in vitro.

## 4. Discussion

In addition to the standard regimen for cervical cancer therapy, treatments with new anticancer agents based on targeting the molecular pathways dysregulated in cervical cancer have emerged as strategies with great potential. Protein kinase CK2 has been shown to be involved in the regulation of cellular and viral proteins relevant for this malignancy [32,33]. For example, in head-and-neck squamous cell carcinoma, CK2 is associated with aggressive tumor behavior and poor clinical outcome, which reinforces the rationale for exploring the use of CK2 inhibitors in the clinical setting [34,35,36]. Recently, it has been shown that CK2 activity is required for the efficient transient and stable replication of various HPV types [37]. Two molecules targeting CK2-mediated signaling, namely, CX-4945/Silmitasertib and CIGB-300 have shown antineoplastic potential and good synergy and/or additivity with cisplatin in cervical cancer treatment [38,39]. The clinically useful effects of the anti-CK2 peptide CIGB-300 have been demonstrated in a phase I/II clinical trial in women with locally advanced cervical cancer [23,24,25]; however, the molecular basis of this clinical effect is still relatively unexplored.

Although the development of CIGB-300 as a potential therapeutic arose from its ability to block HPV-16 E7 phosphorylation, its putative physical interaction with the viral oncoprotein in the cellular context remained to be confirmed. Here, we show for the first time, using in vitro pull-down assays, a clear physical interaction between HPV-16 E7 at a relevant therapeutic dose of CIGB-300. Importantly, that interaction was also confirmed for the E7 protein from the HPV-18 and HPV-11 types, which would support the clinical benefit of CIGB-300 in patients with HPV-18-positive tumors and those with low-risk HPV-infected lesions. To further evaluate the in vivo CIGB-300-E7 interaction between both molecules, we performed in vivo pull-down. We employed HEK293 as an epithelial cell model with high transfection efficiency that has been previously used for studying E6 and E7 interaction partners [40]. Using HEK293 cells overexpressing HPV-16 E7, we observed physical interaction between CIGB-300 and E7 protein. The in vivo binding of the peptide to E7 was also seen in a cervical cell type, confirming the suitability of the HEK293 line for this type of studies.

The in vitro inhibition of HPV-16 E7 CK2-mediated phosphorylation by CIGB-300 has previously been documented [15,41]; here, we examined the in vivo effect of the peptide on E7 phosphorylation using HEK293 cells. Analysis of the phosphorylation of the Ser31/Ser32 phospho-site in E7 after treatment with CIGB-300 showed approximately 40% inhibition after 30 minutes of treatment. Considering that E7 is differentially phosphorylated by CK2 during the cell cycle in the G_1_ phase [42], it remains to be determined whether the inhibitory effect of CIGB-300 on E7 phosphorylation could change according to the cell cycle phase. Recent studies by Basukala et al., using genome editing of cervical cancer-derived C4-1 cells, have shown the relevance of the CK2 phospho-acceptor site in HPV-18 E7 for maintaining a fully transformed phenotype [14]. To further investigate the contribution of E7 phosphorylation inhibition to the cytotoxic effect induced by CIGB-300 in cervical cancer, we exploited CRISPR-edited cells with a mutation within the HPV-18 E7 CK2 phospho-acceptor site. Our data demonstrate that CIGB-300 has a potent dose-dependent cytotoxic effect on C4-1 cells. Consistent with the modest effect of CIGB-300 on E7 phosphorylation, these mutant C4-1 cells did not show a clear difference in the cytotoxic effect mediated by CIGB-300. Taken together, these results indicate that targeting the molecular event of E7 phosphorylation is not a major contributor to the cell death triggered by CIGB-300. However, our data do not rule out that inhibition of E7 phosphorylation by CIGB-300 could be relevant for reducing the proliferative and invasive potential of the transformed cell lines.

Previous studies had indicated that CK2-mediated phosphorylation of E7 is required for pocket protein recognition [10]. The best-characterized E7 ligand is pRB, and an impairment of the E7–pRB interaction has been shown in mutant E7-CK2 phospho-site cell lines [14]. Correspondingly, we wanted to explore the putative effect of CIGB-300 on the interaction of E7 with the tumor suppressor pRB. Using co-immunoprecipitation assays in the HEK293 model, we found a clear decrease of the binding of pRB to E7 after treatment with the CK2 inhibitors CX-4945 and CIGB-300. HPV E7–pRB association abolishes the transcriptional repressor activity of pRB/E2F complexes, causing a dysregulated expression of E2F target genes [43]. Therefore, the antineoplastic effect of CIGB-300 might be supported in part by the targeting of E7 and the rescuing of the tumor-suppressive activity of pRB. Our current studies aim to explore other E7-associated proteins affected by CIGB-300.

## 5. Conclusions

In conclusion, we demonstrated for the first time that CIGB-300 targets E7 proteins from high- and low-risk HPV types. The effect of CIGB-300 on E7 phosphorylation does not appear to have a critical role in the CIGB-300-mediated cytotoxic effect in cervical cancer cells. However, the physical interaction of the peptide with E7 might affect HPV-16 E7 protein function by disrupting its interaction with pRB. Our study reveals novel molecular clues to the mechanism of action of CIGB-300 in cervical cancer.

## Data Availability

All data are already presented in the manuscript and available on request from the corresponding author.
